# Gestational diabetes mellitus placentas exhibit epimutations at placental development genes

**DOI:** 10.1080/15592294.2022.2111751

**Published:** 2022-08-21

**Authors:** Laetitia P. Meyrueix, Raad Gharaibeh, Jing Xue, Cory Brouwer, Corbin Jones, Linda Adair, Shane A. Norris, Folami Ideraabdullah

**Affiliations:** a Nutrition Department, University of North Carolina, Chapel Hill, NC, USA; b Department of Bioinformatics and Genomics, University of North Carolina, Charlotte, NC, USA; c Bioinformatics Service Division, University of North Carolina, Charlotte, NC, USA; d Department of Medicine, Division of Gastroenterology, University of Florida, Gainesville, FL, USA; e Genetics Department, University of North Carolina, Chapel Hill, NC, USA; f Department of Biology and Integrative Program for Biological and Genome Sciences, University of North Carolina, Chapel Hill, NC, USA; g SAMRC Developmental Health Pathways for Health Research Unit, University of Witwatersrand, Johannesburg, South Africa; h Nutrition Research Institute, University of North Carolina, Chapel Hill, NC, USA; i Lineberger Comprehensive Cancer Center, University of North Carolina, Chapel Hill, NC, USA

**Keywords:** Epigenetics, placenta, development, developmental programming, gestational diabetes mellitus, DNA methylation

## Abstract

Gestational diabetes mellitus (GDM) is a maternal metabolic disorder that perturbs placental development and increases the risk of offspring short- and long-term metabolic disorders. The mechanisms by which GDM impairs placental development remain poorly understood. Here, we defined the DNA methylome of GDM placentas and determined whether GDM perturbs methylation at genes important for placental development. We conducted an epigenome-wide association study of 42 placentas from pregnancies in the South African Soweto First 1000 days cohort (S1000). Using genome-wide bisulfite sequencing, we compared non-GDM placentas to GDM placentas with similar proportions from obese and non-obese mothers. Compared to non-GDM, GDM placentas exhibited a distinct methylation profile consisting of 12,210 differentially methylated CpGs (DMCs) that mapped to 3,875 genes. Epigenetically altered genes were enriched in Wnt and cadherin signalling pathways, both critical in placentation and embryogenesis. We also defined regional DNA methylation perturbation in GDM placentas at 11 placental development genes. These findings reveal extensive changes to the placental epigenome of GDM pregnancies and highlight perturbation enriched at important placental development genes. These molecular changes represent potential mechanisms for GDM-induced placental effects that may serve as candidate biomarkers for placental, maternal, and foetal health. Using a study design that used similar proportions of obese and non-obese mothers in our case and control pregnancies, we minimized the detection of changes due to obesity alone. Further work will be necessary to investigate the extent of the influence of obesity on these GDM-related placental epigenetic changes.

## Introduction

Gestational diabetes mellitus (GDM) rates are increasing globally, with an estimated 14% of pregnancies affected in 2021^1^. Despite a growing body of research on GDM world-wide, there are only limited data on the prevalence and aetiology of GDM in African countries. The current estimate for the continent of Africa is 14.2%[1], while South Africa’s GDM rates are estimated at 9.1%[2].

GDM is defined as glucose intolerance identified for the first time during pregnancy and is diagnosed at 24 to 28 weeks of gestation [3] in women not previously diagnosed with Type 1 or Type 2 diabetes mellitus. In most cases, GDM is caused by pancreatic β-cell dysfunction [4], which leads to chronic insulin resistance [5] and subsequent hyperglycaemia[6]. Risk factors for GDM include maternal obesity [7], history of delivering a child with macrosomia [8], older maternal age [9], and family history of diabetes [10]. Ethnicity has also been proposed as a risk factor for GDM [11,12], but this has not yet been investigated in a global context.

GDM increases the risk for obstetric complications that pose a risk to foetal health, such as pre-eclampsia [13], operative delivery [14], macrosomia [14], and neonatal hypoglycaemia[15]. Offspring of GDM pregnancies are at increased risk of obesity [16] and impaired glucose tolerance [17] in childhood, and at increased risk of hypertension [18], metabolic syndrome [19], and Type 2 diabetes [20] in adulthood. Molecular mechanisms underlying these effects are unclear. However, a growing body of data suggests a role for placental defects [21]. Although candidate molecular targets for the diagnosis and intervention of the effects of GDM on offspring have been identified in the placenta [22], their roles in placental health remain unclear and predictive biomarkers of foetal health have not been sufficiently defined for clinical use.

The placenta functions as the sole transporter for foetal nutrients, gasses, and waste products. It also acts as a barrier to harmful maternal exposures. Poor maternal metabolic health has been shown to alter placental morphogenesis, function, and pathology, and subsequently impair foetal development [23,24]. Therefore, placental dysfunction has a direct impact on foetal health. GDM has been linked to impaired placenta development manifested as increased placenta size [21,25] and angiogenesis [25,26]. These GDM-induced placental defects are often seen in tandem with adverse foetal outcomes, including foetal macrosomia, large-for-gestational-age, and impaired glucose tolerance [16,20,27]. This suggests GDM-induced impaired placental development plays a vital role in poor foetal and neonatal outcomes. Genes regulating these defects could serve as important biomarkers to predict health outcomes across the lifespan [28].

Placental development and function are tightly regulated by epigenetic mechanisms [29]. DNA methylation is one of the many epigenetic mechanisms [30] that together control gene expression, genomic stability, and normal development [31]. Dysregulation of placental epigenetic mechanisms impairs placental development and subsequent functions required for optimal foetal health. Placental DNA methylation is essential for placentation and diffusional permeability capacity [32–34]. GDM has been proposed to alter placental function by perturbing placental epigenetic mechanisms. For example, placentas from GDM pregnancies exhibit altered methylation at placental energy metabolism genes, such as *LEP, ADIPOQ, LPL, and PPARγ* [35–38]. However, the effects of GDM on placental development genes remain unclear [39].

The current study describes our assessment of the impact of GDM on genome-wide DNA methylation patterns in placentas from a South African pregnancy cohort. We focus on the perturbation of placental development genes. Studies show that obesity influences pregnancy insulin levels and thus GDM [40]. We designed this study to assess the impact of GDM while minimizing the effects caused by obesity alone. We conducted our experiment using genome-wide bisulfite sequencing (bis-seq) with the Agilent SureSelect Methyl-seq target enrichment system, which covers CpG islands, shores/shelves (±4kb), GENCODE promoters, enhancers, regulatory regions, and DNase I hypersensitive sites. Unlike array-based methods, genome-wide sequencing allows us to assess consecutive CpGs in regulatory regions within gene bodies and between intergenic regions.

## Materials and methods

### Sample population

Forty-two de-identified placenta samples and data were obtained from the Soweto First 1000 Days Pregnancy Cohort (S1000, SAMRC Developmental Pathways for Health Research Unit at the University of Witwatersrand) [41]. The S1000 cohort was recruited from the Antenatal Clinic and Fetal Medicine Unit at Chris Hani Baragwanath Academic Hospital from June 2013 through July 2016. S1000 participants were Black indigenous South Africans living in Soweto, ≥18 years of age, 14–20 weeks pregnant with singleton pregnancies. Women with known pre-pregnancy diabetes or pregnancies with maternal or foetal complications were excluded. 11.2% of the 741 women tested for GDM [3] were diagnosed with GDM. The current study selected 42 samples using the criteria: HIV-negative, 21 GDM and 21 non-GDM samples, and a similar proportion of obese and non-obese mothers in each disease category. At the time of GDM diagnosis, three non-GDM and two GDM mothers of these selected placenta samples exhibited hypertension with systolic blood pressures ranging from 140 to 142 mmHg and/or diastolic blood pressure ranging from 90 to 93 mmHg. Three non-GDM and five GDM mothers exhibited anaemia with haemoglobin levels ranging from 8 to 10 g/dL. Of the 21 samples from GDM pregnancies, eight received lifestyle intervention, and four received a combination of lifestyle intervention and metformin. None were on insulin.

### Placenta sampling

Placental weight, neonatal weight, length, and head circumference were measured at birth. Placenta samples were collected within 1 h of delivery, flash frozen in liquid nitrogen, and stored at −80°C until use. After excess blood was removed, 10 mm tissue punches were taken from the foetal side of the placental disc, at least 3 cm from the placenta edge, and avoiding the umbilical cord and any visible lesions or abnormalities [42].

### Epigenome-wide association study (EWAS)

Placental samples were genotyped using PCR amplification of *SRY* to confirm the sex of the offspring. Genomic DNA was extracted from placenta samples using phenol-chloroform as previously described [43]. The DNA quantity and purity were assessed using the Nanodrop 2000 spectrometer (Thermo Scientific), and the quality of double-stranded DNA (dsDNA) was measured using the Quant-it PicoGreen dsDNA assay (#P7589, Life Technologies). Three μg dsDNA was used for library preparation with the SureSelectXT Human Methyl-Seq Target Enrichment Kit (Agilent) following the manufacturer’s instructions. Libraries were multiplexed and sequenced on the Illumina HiSeq25000 (Illumina) at the David H. Murdock Research Institute (DHMRI). Illumina HiSeq single-end 100 bases long reads were filtered for adaptors and quality trimmed using Trimmomatic [44] (v. 0.35) with a sliding window of four bases and a minimum quality of 15 (Phred score ≥15). Reads were mapped to the UCSC hg19 human genome, and methylation calls were made using Bismark [45] (v. 0.14.5) utilizing Bowtie2 [46] (v. 2.2.5). For quality control, we only used CpGs with ≥10X coverage and ≥20 quality score [47]. Single nucleotide polymorphisms (SNPs) were identified using the UCSC hg19 dbSNP150 dataset and removed to limit false methylation calls. CpGs in ‘blacklist’ regions annotated by ENCODE were also removed from the dataset as they represent repetitive or poorly annotated regions that cannot be accurately aligned [48].

Differentially methylated CpGs (DMCs) were determined using methylKit [49] (v. 1.5) in R [50] (v. 3.4.1). As we previously described [47], we identified CpGs that were differentially methylated between GDM and non-GDM groups using the methylKit [49] logistic regression analyses, which compared the proportion of methylated versus unmethylated cytosines among the reads at a given CpG locus. In the regression model, we adjusted for sex of offspring, maternal BMI, gestational age, maternal age, and mode of delivery. The effect size (weighted by read coverage) was calculated as the mean methylation difference between non-GDM and GDM. The SLIM method was used in methylKit to correct for multiple testing [49] and an FDR (q-value) <0.01 was considered significant.

### Annotation of differentially methylated CpGs, pathway analyses, and annotation of regulatory regions

Gene annotation of DMCs was performed using Hypergeometric Optimization of Motif EnRichment [51] (HOMER) (v. 4.10), which annotates in order: 1. TSS (defined from −1kb to +100bp), 2. TTS (defined from −100 bp to +1kb), 3. Exons, 4. 5'UTR Exons, 5. 3'UTR Exons, 6. Introns, and 7. Intergenic. Pathway analysis was completed using Protein Analysis through Evolutionary Relationships [52,53] (PANTHER) (v.16.0, released 2020–12-01) overrepresentation test (Fisher’s exact) and false discovery rate tools. Annotations for regulatory elements were performed using the ORegAnno database through the UCSC Genome Browser (hg19). Using bedtools [54], we identified 4,942 DMCs at ORegAnno regulatory regions, regulatory polymorphisms, transcription factor-binding sites, regulatory haplotypes, and miRNA binding sites.

### Quantitative real-time PCR (qRT-PCR)

#### Statistical analyses

All statistical analyses and plotting were completed using R Software (v.4.0–4.1.1). Graphics were made using the R core package and packages gplots [55], ggplot2 [56], and RColorBrewer [57]. Wherever applicable, normality of the data was confirmed by quantile plot and Shapiro-Wilk goodness-of-fit test, and equal variance was tested by Bartlett’s test. For data not normally distributed, we normalized by log transformation (*EBF1, HIST1H3E*, and *SMOC2* expression levels). Covariates for all models were determined by testing the association of the primary outcomes with maternal (e.g., maternal age, parity, mode of delivery, maternal BMI) and foetal characteristics (e.g., gestational age, sex) using a t-test (categorical covariates) or Spearman’s correlation (continuous covariates). All multivariate linear regressions were adjusted for maternal age, gestational age, maternal BMI, mode of delivery, and sex of offspring (except for the expression analysis, which did not include mode of delivery).

Differences in maternal characteristics between non-GDM and GDM were tested using t-test or chi-square test. Multivariate linear regression was utilized to test the association between (i) GDM status and placental & birth outcomes with and without stratification by sex [female data not shown due to small GDM sample size]; (ii) GDM status or sex of offspring with mean methylation of all queried CpGs (n = 989,582) and mean methylation of all chr X queried CpGs (n = 20,707); (iii) GDM status and mean regional methylation levels; and (iv) GDM status and expression levels. Pearson Chi-square was used to test the null hypotheses that (i) proportions of GOM and LOM DMCs are the same across the chromosomes; (ii) the proportions of genic locations are the same across effect size, and (iii) the proportions of GOM and LOM DMCs are the same across effect size. Genic location was categorized as intragenic, intergenic, promoter-TSS, and TTS. Fisher’s exact test was used to test the null hypotheses that (i) the proportions of intragenic versus intergenic CpGs are the same between the DMCs and the total queried CpGs and (ii) the proportions of genic locations are the same across effect size (similar results achieved with the chi-square test). The expected number of DMCs per chromosome was calculated by multiplying the number of queried CpGs on each chromosome to the ratio of total DMCs (12,210)/total number of queried CpGs (989,582).

The PCA plot was calculated using *prcomp* in R with a singular value decomposition of the centred data matrix. The unsupervised hierarchical clustering and heatmap were created utilizing Euclidean distance and complete linkage.

Regional methylation of the candidate genes was calculated using the mean methylation level of all CpGs located between the defined flanking DMCs for each genic region (e.g., promoter-TSS, intron 1, etc.). The Benjamini-Hochberg (BH) method was used to correct for multiple testing [58].

## Results

### Characteristics of 42 mother-infant dyads from the S1000 cohort

Placentas from 42 pregnancies were selected from the Soweto First 1000 Days (S1000) cohort. We used a case–control study design to compare GDM pregnancies to non-GDM pregnancies, selecting for a similar proportion of obese and non-obese mothers in each disease category (*see Methods*). Table 1 describes the cohort characteristics from the 42 selected pregnancies. As we previously reported [59], the selected GDM pregnancies had higher maternal age, maternal parity, and proportion of male offspring compared to non-GDM.

**Table 1.** Characteristics of the 42 mother-infant dyads from the S1000 days cohort.

	*nonGDM (n = 21)*		*GDM (n = 21)*		
Cohort Characteristics	Mean ± SD/(%) of total	Range	Mean ± SD/(%) of total	Range	p-value
Maternal BMI^1^ (1^st^ trimester, kg/m^2^)	28.2 ± 7.5	18.2–48	30.7 ± 5.8	19–46.5	0.226
Maternal Obesity Status^2^ (% with obesity; BMI>30)	38%		52%		0.352
Maternal Age^1^ (years)	27.6 ± 4.9	19–37	32.8 ± 5.2	21–43	0.002**
Gestational age^1^ (weeks)	38.7 ± 1.6	35–41	38 ± 2.4	33–41	0.263
Parity^1^ (# of full-term pregnancies)	0.81 ± 0.7	0–2	1.6 ± 0.9	0–4	0.003**
Mode of Delivery^2^ (% caesarean)	24.4%		34.2%		0.279
Sex^2^ (% female)	14 F, 7 M (67%)		2 F, 19 M (10%)		0.0001**

^1^two-tailed t-test; ^2^chi-square.

To determine the relationship between GDM status and offspring birth and placenta outcomes, we assessed differences in placenta weight (grams), placenta efficiency (the ratio of foetal to placental weight), birth weight (grams), birth length (cm), birth head circumference (cm), and ponderal index. After adjusting for maternal age, gestational age, maternal BMI, and mode of delivery, we detected significantly lower birth weight in GDM-exposed males compared to non-GDM-exposed males (Supplemental Table S1). There were not enough female GDM samples to independently test the effects in females. However, when male and female samples were combined, there was no significant association between GDM and birth weight (Supplemental Table S1), suggesting female offspring may respond differently than males. No significant associations were detected for any other outcomes assessed, consistent with a lack of postnatal changes observed in the greater S1000 cohort [41].

### S1000 placentas from GDM pregnancies exhibit genome-wide epigenetic dysregulation

To determine the extent to which GDM alters the placental methylome, we measured genome-wide DNA methylation through bisulfite sequencing (bis-seq) using the Agilent SureSelect Methyl-seq target enrichment system. After the removal of data that did not pass quality controls (see Methods), 989,582 CpGs were queried.

**Figure 1.**
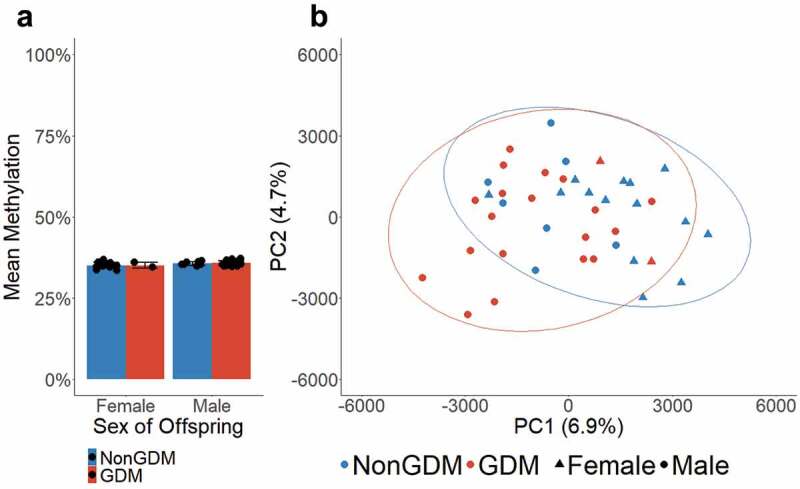
Global mean methylation of S1000 placenta samples. (a) Global mean methylation of placenta samples separated by sex of offspring and bar colours indicate GDM status. Error bars show standard deviation. (b) PCA plot of all queried CpGs (n = 989,582), colours indicate GDM status, shapes indicate sex, ellipses indicate 95% confidence intervals.

Previous studies have reported an association between mean placental methylation and GDM status [60] as well as mean placental methylation and sex of offspring [61]. Using the average methylation of all queried CpGs (n = 989,582) for each sample, we determined that the mean genome-wide methylation of S1000 placentas did not differ by GDM status (p = 0.500) or sex of offspring (p = 0.082) (Figure 1a). Additionally, we tested whether male vs. female offspring, which carry one versus two X chromosomes, respectively, exhibited differences in mean methylation on the X chromosome (n = 20,707 CpGs). The mean methylation of the X chromosome did not differ by offspring sex (p = 0.409) or GDM status (p = 0.988) (Supplemental Figure S1). Principal component analysis (PCA) was performed as a secondary measure to assess whether methylation levels at all queried CpGs differed between GDM and non-GDM. Although the PCA plots showed some clustering by GDM status (Figure 1b), samples did not cluster by offspring sex (Supplemental Figure S2).

**Figure 2.**
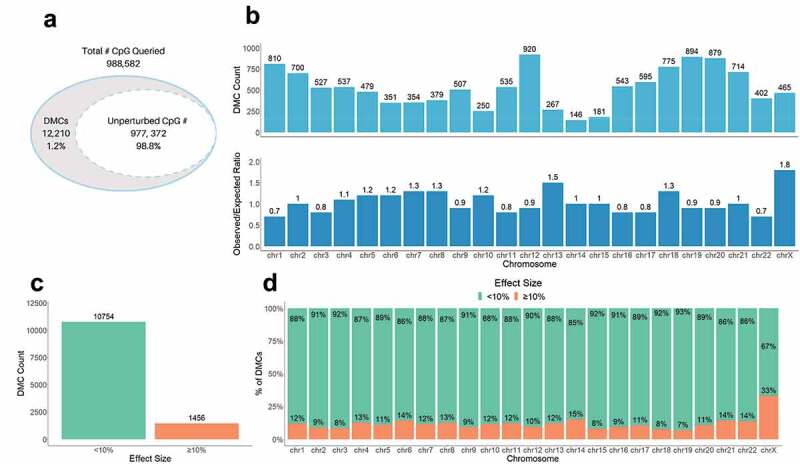
Genome-wide difference in GDM placental methylation status compared to non-GDM. (a) Proportion of total CpGs queried with no changes (unperturbed) vs. significant changes in methylation (DMCs) between GDM and non-GDM placentas; (b) Distribution of DMCs across the genome. Top graph shows number of DMCs (DMC count) localized to each chromosome. Bottom graph shows the ratio of observed/expected DMCs by chromosome; (c) Number of DMCs with effect size <10% vs. ≥10% (d) Proportion of DMCs with different effects sizes (<10% vs. ≥10%) on each chromosome.

We used the *R* package methylKit [49] to identify CpGs with significant differences in methylation levels between non-GDM and GDM placental samples (using FDR <0.01). We found 12,210 differentially methylated CpGs (DMCs) distributed across the genome (Figure 2a), implicating that GDM effects on placental methylation are genome-wide. To examine if these changes were chromosome-specific (implicating a locus targeted or enriched mechanism of epigenetic perturbation), we calculated the relative enrichment of DMCs at each chromosome as the ratio of the actual and expected number of DMCs using the proportion of queried CpGs on each chromosome (Figure 2b). Several chromosomes exhibited overrepresentation of DMCs, most notably, chromosome (chr) X and chr 13, with 1.8- and 1.5-fold enrichment in observed DMCs, respectively. Additionally, several chromosomes exhibited underrepresentation, most notably, chr 1 and chr 22 (both 0.7-fold). This shows that GDM-associated methylation changes are not evenly distributed across the genome, which could reflect the enrichment of perturbed genes on these chromosomes.

**Figure 3.**
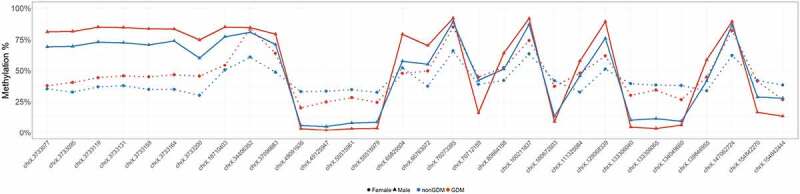
Sex-specific methylation levels of GDM-related large effect size DMCs (>20%) on chr X. Percent methylation at DMCs with effect sizes >20% on chr X. Mean % methylation shown for GDM vs nonGDM samples (red vs blue) stratified by sex (circle vs triangle). DMCs shown are not consecutive or drawn to scaled distance.

To better characterize methylation changes related to GDM, we categorized GDM-associated DMCs based on the effect size of the change in methylation between GDM and non-GDM samples. We found that ~12% of the DMCs (1,456) exhibited a substantial difference in DNA methylation, ≥10% (Figure 2c). These larger ≥10% changes were distributed across all chromosomes, with chr X exhibiting the greatest number and proportion of the ≥10% changes (compared to autosomes, p-value = 7.2e-35) (Figure 2d). An assessment of the large effect size DMCs (>20%) on chr X confirmed that although the overall methylation status differed between males and females [61,62], the extent and direction of change caused by GDM were similar for both sexes (Figure 3).

To test for consistency in the directionality of GDM-related placental DNA methylation changes (indicative of a common mechanism of perturbation such as global hyper or hypomethylation), we assessed whether DMCs represent a gain (GOM) or loss of methylation (LOM). We detected a similar proportion of total GOM and LOM GDM-associated DMCs across the genome (Supplemental Figure S3a). However, the proportion of GOM and LOM differed by the extent of methylation change (with changes ≥10% more likely to be GOM, p-value = 7.81e-26, Supplemental Figure 3A) and chromosomal location (p-value = 1.46e-29, Supplemental Figure S3b).

### GDM-associated DMCs are localized predominantly within gene bodies and gene regulatory regions

**Figure 4.**
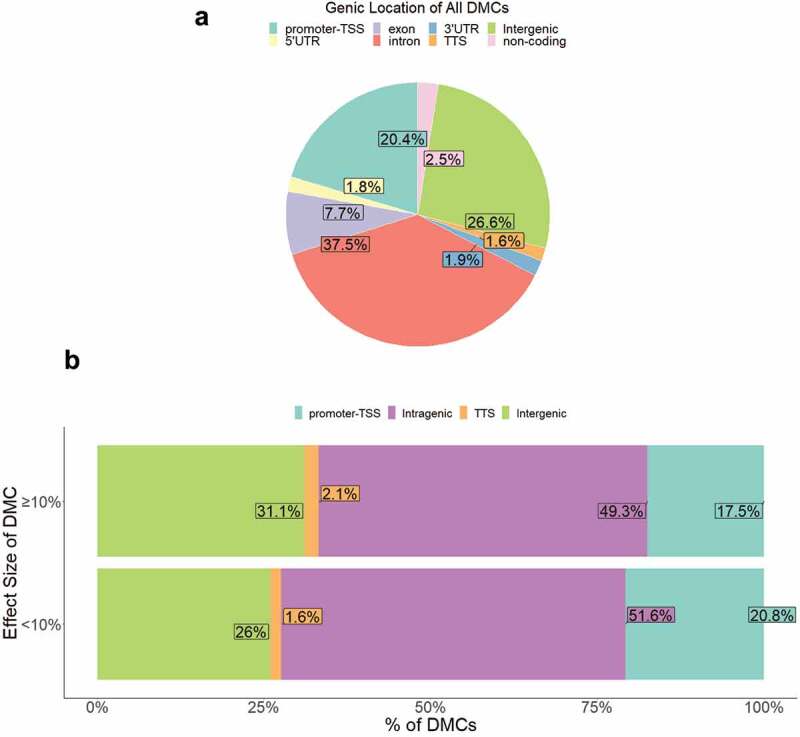
Genic location of DMCs. (a) Proportion of DMCs localized to each genic region shown. (b) Proportion of DMCs localized to each genic region separated by effect size <10% vs. ≥10%.

To investigate the potential functional (e.g., gene expression) relevance of the DMCs identified, we first categorized DMCs by localization in either: (1) intragenic & proximal regulatory (PR) regions including promoter regions (≤1kb upstream or ≤100 bp downstream of the promoter-transcription start site, TSS) and transcription termination regions (≤100 bp upstream or ≤1kb downstream of the transcription termination site, TTS); or (2) intergenic regions (≥1kb upstream of the TSS or downstream of the TTS). Approximately 73% of DMCs were located in the defined intragenic & PR regions, ~53% of which were intronic (Figure 4a). Compared to the queried CpGs, which were already enriched for intragenic & PR regions (~81%), the GDM-associated DMCs were annotated to a significantly lower proportion of intragenic & PR regions (73%) (p-value = 9.2e-97).

We then examined whether effect sizes of GDM-related DMCs differed by genic location to assess whether specific gene regulatory regions exhibited more or less perturbation. Genic location of DMCs varied by effect size such that medium-to-large effect size DMCs (≥10% change in methylation) occurred less frequently at intragenic & PR regions (~69%) compared to small effect size (<10%) DMCs (~74%) (p = 5.0 x10^−5^) (Figure 4b). The directionality of methylation change was similar between genic locations (Supplemental Figure S3c).

Of the 12,210 GDM-related DMCs, 4,942 were annotated to ORegAnno transcription factor-binding sites and regulatory regions. Supplemental Table S2 lists the top 10 transcription factors that DMCs are localized to, with ~50% (2,490) localized to EGR1, SMARCA4, or RBL2 binding sites.

### Placental DNA methylation profiles cluster by GDM status

**Figure 5.**
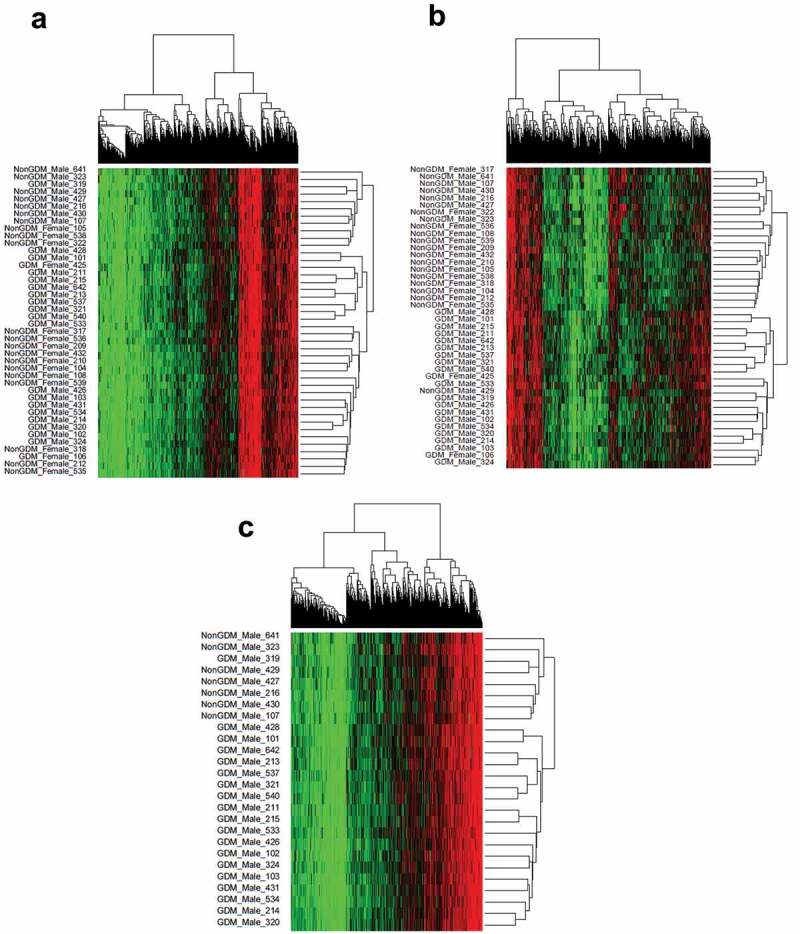
Sample methylation profiles at GDM-related DMCs using hierarchical clustering. (a) Hierarchical clustering of DMCs (n = 12,210) in all samples (n = 42); (b) Hierarchical clustering of DMCs with ≥10% effect size (n = 1,456) in all samples (n = 42); (c) Hierarchical clustering of DMCs (n = 12,210) in male samples (n = 26).

To evaluate whether the combined methylation status (methylome) at GDM-related DMCs forms a consistent methylation profile of GDM disease status, we conducted unsupervised hierarchical clustering analyses of the DMCs. Considering all 42 male and female samples and all DMCs regardless of effect size, there was no distinction between GDM and non-GDM samples (Figure 5a). This finding is not surprising considering that this method does not account for confounding variables that may contribute to small and inconsistent methylation changes among the samples. However, the hierarchical clustering of all samples utilizing only DMCs with ≥10% methylation changes (n = 1,456) did show a distinct grouping by disease status (Figure 5b). Thus, DMCs ≥10% represent a more reproducible measure of GDM effects on the placental methylome. When the male samples alone were analysed for all 12,210 DMCs regardless of effect size, placental methylation profiles clustered by GDM status (Figure 5c). These data demonstrate that male GDM placentas exhibit a distinct methylome at DMC loci that differs from non-GDM samples. In contrast, females did not exhibit similar clustering for all 12,210 DMCs (Supplemental Figure S4), possibly due to the small sample size (n = 2) of the GDM female group.

### Placental development genes are epigenetically perturbed in S1000 GDM placentas

To investigate whether GDM-induced methylation changes are enriched in gene pathways important for placental development, we defined the gene ontology categories for genes with proximity to DMCs. Of the 12,210 DMCs, 8,962 annotated to 3,875 unique genes. Using the PANTHER gene ontology consortium, we identified 3,278 DMC-associated genes (DMCs in the gene body or within 1 kb upstream of the TSS). The top 5 pathways were Wnt signalling, cadherin signalling, gonadotropin-releasing hormone receptor, heterotrimeric G-protein signalling, and angiogenesis pathways (Supplemental File S1). Using statistical overrepresentation analysis in PANTHER, we identified significant enrichment for pathways involved in cadherin signalling, Wnt signalling, metabotropic glutamate receptor group III, ionotropic glutamate receptor, and heterotrimeric G-protein signalling pathway-Gi alpha and Gs alpha mediated (Table 2). Cadherin signalling and Wnt signalling pathways remained significant when the analysis was performed with DMCs of effect size ≥10% (Supplemental Table S3).

**Table 2.** Overrepresented PANTER pathways associated with DMCs.

PANTHER Pathway	# of Genes	P-value	FDR	Fold Enrichment
Cadherin signalling pathway (P00012)	63	4.09E-08	3.41E-06	2.37
Wnt signalling pathway (P00057)	95	5.80E-07	3.23E-05	1.85
Metabotropic glutamate receptor group III pathway (P00039)	32	5.16E-06	2.15E-04	2.82
Ionotropic glutamate receptor pathway (P00037)	24	5.44E-05	1.51E-03	2.96
Heterotrimeric G-protein signalling pathway-Gi alpha and Gs alpha mediated pathway (P00026)	53	4.77E-05	1.59E-03	1.97

A total of 3,875 genes were inputted into PANTHER and 3,278 of them mapped to the PANTHER database. Overrepresentation was calculated using a Fisher’s exact test and the False Discovery Rate was calculated.

### Placental genes that regulate hormone signalling, growth, and energy metabolism exhibit GDM-related regional changes in DNA methylation that may serve as candidate biomarkers of placental injury

To identify epigenetically altered genes most likely to play a role in placenta development and thus serve as candidate epigenetic markers of GDM-related placental injury, we used sequential ranking criteria to define placental development genes with the most evidence for epigenetic dysregulation. First, 62 genes were selected based on the criteria of genes with at least three DMCs (at least 1 with ≥10% effect size) and known role(s) in mouse placental or embryonic development (Supplemental File S2). Next, the top 14 GDM biomarker candidates were selected based on previous evidence of perturbation of placental methylation or expression (Table 3). These genes have known or predicted roles in growth, morphogenesis, and vascularization (*FGF18* [63], *ESX1* [64], *WNT2* [65], and *SMOC2* [66]); cell proliferation and differentiation (*NTN1* [67] and *GATA4* [68]); adipogenesis and energy metabolism (*EBF1* [69], *PPARGC1A* [70], and *PDXK* [71]); genome organization (*HIST1H3E* [72]); insulin regulation (*PTPRN2* [73], ADORA2B [74], and *IRS1* [75]); and hormone signalling (*MKRN3* [76]).

**Table 3.** Summary of 14 candidate genes.

Gene Name	Chr #	Total # of DMCs	# of DMCs ≥10%	Direction of Change for DMCs (% GOM)	# of DMCs in TFBS or RR	Demonstrated mechanistic evidence in model systems?		Previous evidence of link in human placenta?		Previous evidence of link to GDM?
						Role in placenta development?	Role in embryonic development?	Changes in placental methylation?	Changes in placental expression?	
HIST1H3E	6	22	20	83%	22				✓	
PTPRN2	7	135	19	16%	25			✓		
SMOC2	6	64	11	98%	3				✓	
EBF1	15	8	7	100%	0		✓		✓	
NTN1	17	11	7	100%	9				✓	✓
PDXK	21	7	6	86%	6			✓		
ADORA2B	17	7	4	100%	7			✓	✓	✓
GATA4	8	8	4	50%	0		✓	✓	✓	✓
IRS1	2	10	4	100%	4				✓	✓
PPARGC1A	4	5	3	0%	5		✓	✓		✓
WNT2	7	5	2	100%	0	✓	✓	✓	✓	✓
ESX1	X	3	1	33%	0	✓			✓	
FGF18	5	11	1	100%	2		✓			
MKRN3	15	10	1	0%	8					

Candidate genes were selected from 3,875 unique genes shown to have differentially methylated CpGs (DMCs) with larger methylation changes and most likely to play a role in placenta development. GOM represents a gain of methylation in the GDM group. Transcription factor binding site (TFBS) and regulatory region (RR).

For each candidate gene, we assessed the regional change in methylation across consecutive CpGs to identify significantly differentially methylated regions (DMRs) between GDM and non-GDM samples. We identified 28 GDM-related DMRs at 11 candidate genes (Figure 6, Supplemental File S3). *PDXK, HIST1H3E*, and *SMOC2* exhibited increased promoter methylation for GDM placentas (Figure 6a). In contrast, *PPARGC1A* and *MRKN3* exhibited decreased promoter methylation for GDM placentas (Figure 6b). Several other genes exhibited altered methylation within the gene body in GDM placentas. *ADORA2B, IRS1, and NTN1* all exhibited increased methylation in GDM samples for at least one exonic DMR (Figure 6c). *EBF1* and *FGF18* exhibited increased methylation and *PTPRN2* exhibited decreased methylation in GDM samples at intronic DMRs (Figure 6d). Despite the identification of several consecutive DMCs associated with *GATA4, ESX1*, and *WNT2*, the mean combined CpG methylation for these genes was not significantly different (Figure 6e, Supplemental File S3). For *ESX1* and *GATA4*, this was likely caused by differences in the directionality of DMCs across the region. This finding does not reduce the importance of these DMCs nor the potential impact on related genes, but instead indicates that the molecular targets at these genes, for the purpose of biomarkers, will need to be CpG specific as opposed to regional.**Figure 6.**
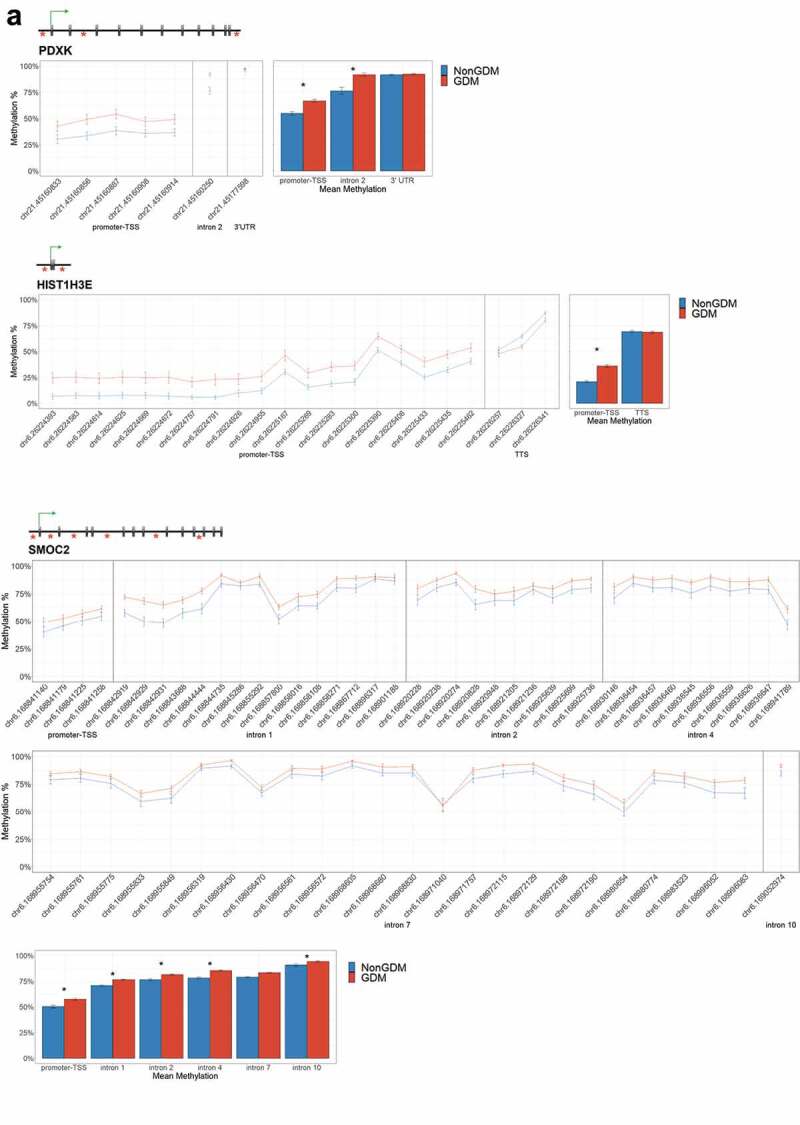
DMRs at eleven candidate genes of GDM-related placental injury. Line graphs show mean sample DNA methylation levels (± standard error of the mean, SEM) across consecutive DMCs for GDM (red) vs non-GDM (blue) samples (N = 42 total). Bar graphs show mean CpG methylation (± SEM) across the individual genic regions shown in the line graph for GDM vs. non-GDM samples. (a) Genes with higher regional promoter methylation levels in GDM samples: *PDXK*, SMOC2, and *HIST1H3E*; (b) Genes with lower regional promoter methylation levels in GDM samples: *PPARGC1A* and *MKRN3*; (c) Genes with higher regional exonic methylation in GDM samples: *ADORA2B, IRS1, NTN1*; (d) Genes with altered regional intronic methylation in GDM samples: *EBF1, FGF18, PTPRN2*; (e) Genes with altered consecutive DMCs but no significant mean regional change: *ESX1, WNT2, GATA4*. Asterisks indicate FDR<0.05 after correction by BH method.

**Figure 6.**
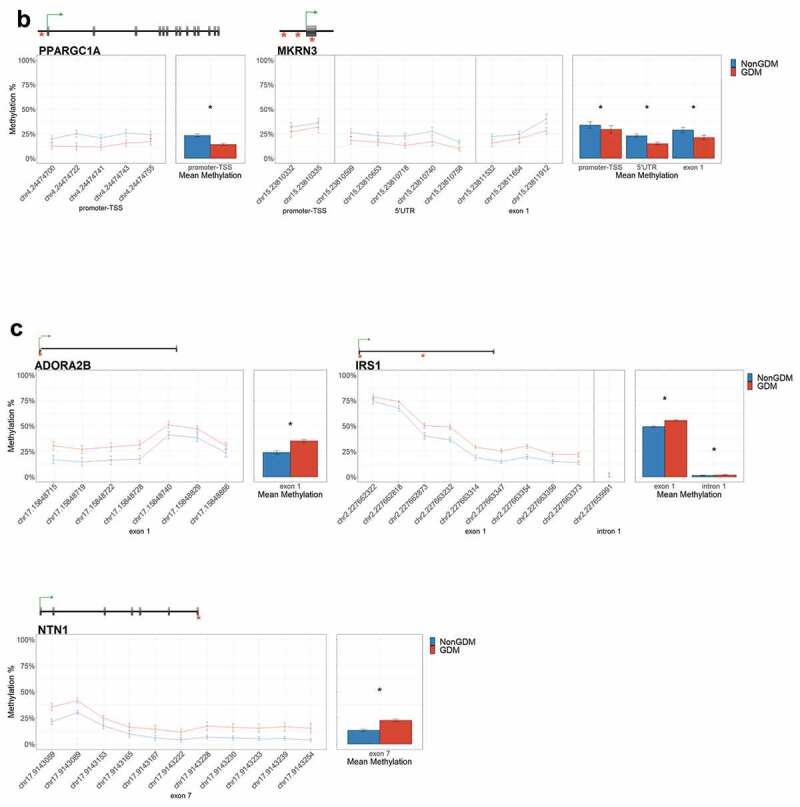
(Continued).

**Figure 6.**
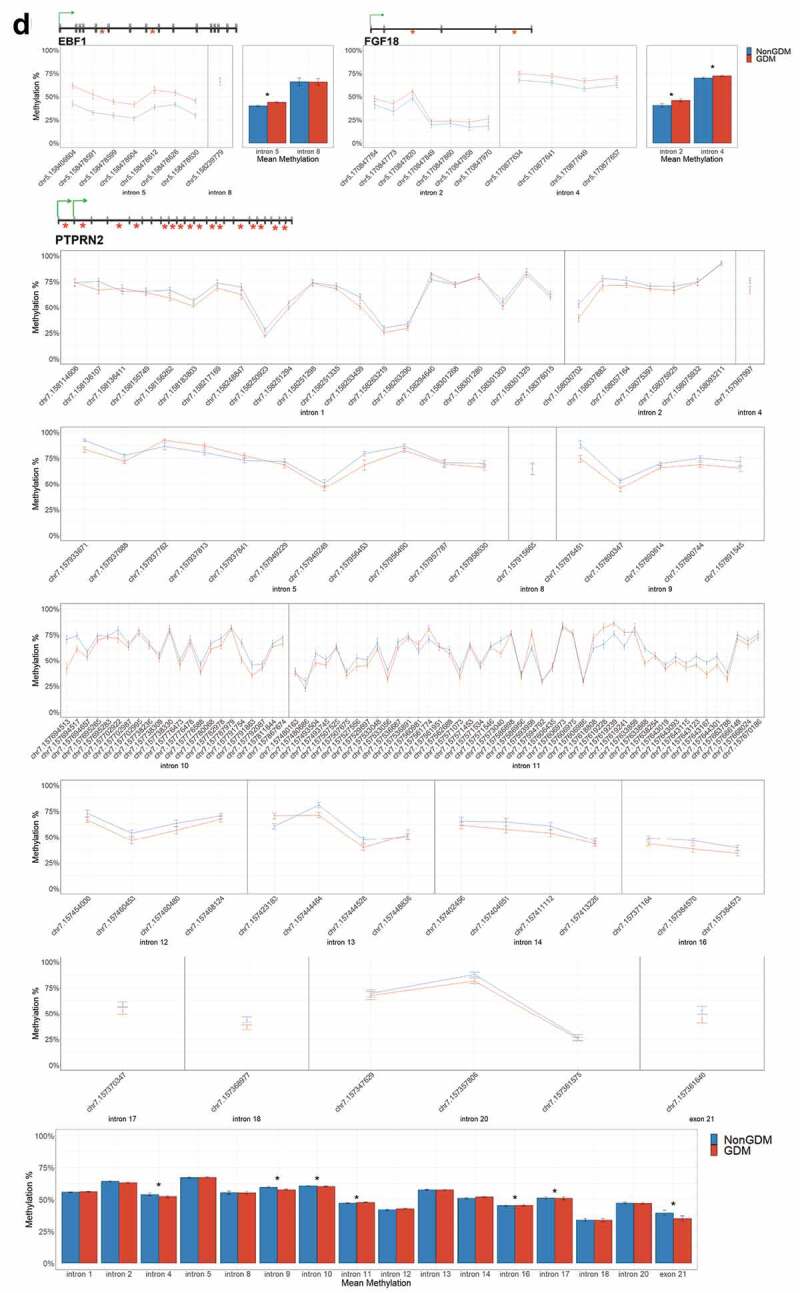
(Continued).

**Figure 6.**
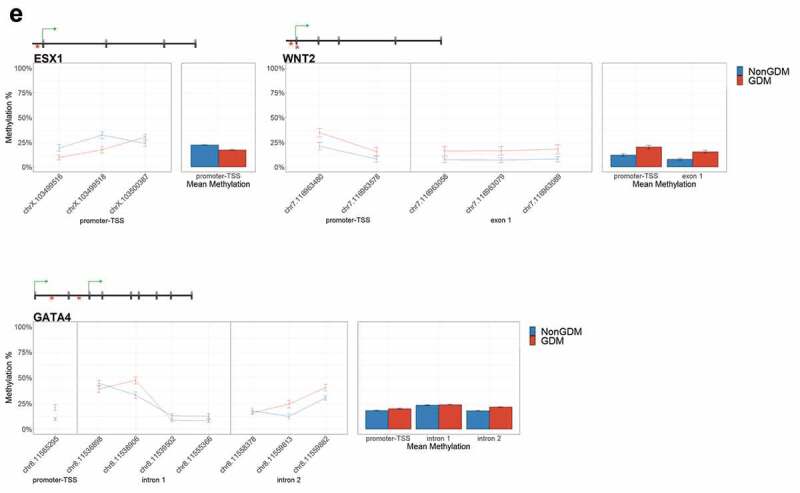
(Continued).

**Figure 7.**
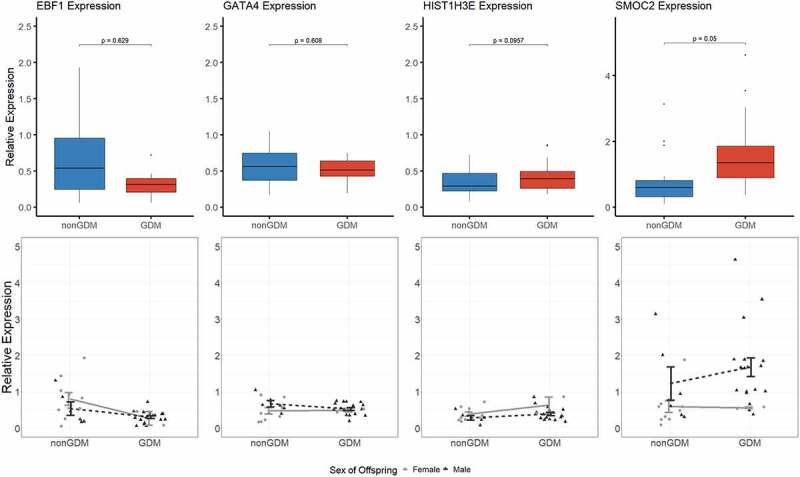
Candidate gene expression levels. Top panels show boxplots of the relative expression levels of four selected candidate genes, *EBF1, GATA4, HIST1H3E*, and *SMOC2*. P-values calculated by linear regression analyses with adjustment for maternal age, maternal BMI, sex of offspring, and gestational age. Bottom panels show individual methylation levels for each male (triangle) or female (circle) placenta sample (N = 36 total) and the line shows the difference in mean methylation (± SEM) of male (black line) GDM vs nonGDM samples (± SEM) or female (grey line) GDM vs non-GDM samples.

Due to limited sample availability, we were unable to detect expression changes in the S1000 term placenta samples at all the candidate epigenetic biomarkers of GDM-related placental injury. Transcript levels were measured at four candidate genes (*EBF1, GATA4, HIST1H3E*, and *SMOC2*) selected based on reproducible detectable transcript levels in the S1000 full-term placentas. After adjustment for maternal age, maternal BMI, sex of offspring, and gestational age, *SMOC2* exhibited the most promising difference in expression between GDM and non-GDM samples (p = 0.050) (Figure 7). However, none of the changes were statistically significant (Figure 7).

## Discussion

Our study shows that GDM is associated with genome-wide perturbation of the term placental methylation. DNA methylation profiles formed by 12,210 DMCs were sufficient to cluster samples by disease status. Almost two-thirds of these epimutations were located within a gene body or proximal regulatory region (e.g., promoter) with enrichment at placental development genes, including those involved in Wnt and cadherin pathways. We identified and characterized GDM-related epigenetic changes at 11 genes that could serve as candidate biomarkers of placental injury. These findings further our understanding of the role of GDM in placental development while also providing a novel assessment of the effects in a South African population, a historically underrepresented and understudied African population.

This is the first study to examine the effects of GDM on placental epigenetic mechanisms in pregnancies from an African population. In addition, a major strength of this study was our use of bisulfite sequencing to assess the effects of GDM on the human placental methylome. Prior studies have assessed similar effects in populations from Canada [77–80], United States of America [81,82], United Kingdom [83], and China [84,85]. However, these previous studies utilized genome-wide methods that are either limited in genome coverage cannot assess regional changes, or are limited to restriction enzyme sites. Findings from this current study provide a unique opportunity to characterize the impact of GDM more thoroughly across the placental methylome while elucidating its effect on a genetically and geographically different population. In addition, another strength of this study is that we included a similar proportion of non-obese and obese in the non-GDM and GDM groups to minimize the effect of obesity alone while also adjusting for maternal BMI in our regression analysis.

GDM placentas exhibited a change in DNA methylation enriched at genic regions. Of 12,210 DMCs identified, ~73% were located either in intragenic regions or within 1kb of the TSS (promoter) or TTS. Although DNA methylation is present across the genome in both inter- and intra-genic regions, methylation changes within regulatory regions such as the promoter or gene body are often associated with altered transcription and subsequent protein levels [86–88]. As is common for term placentas [89], a limitation of our study is that we were unable to assess genome-wide placental transcription due to poor RNA integrity in a majority of samples. Targeted transcriptional assessment of four candidate genes with DMRs (*EBF1, GATA4, HIST1H3E*, and *SMOC2)* did not reveal significant changes in transcript levels. The GDM-related DNA methylation changes identified in S1000 term placentas may represent residual signatures of transcriptional activity during earlier stages of development and not the current transcriptional state [90]. Alternatively, these epigenetic perturbations alone may not be sufficient to dysregulate gene expression. Future work to assess GDM-related transcriptional changes at all of the candidate genes, with a larger set of samples and if possible at earlier developmental stages will help address this question.

The lack of detection of transcriptional dysregulation of epigenetically altered genes in GDM placentas does not negate the importance of DNA methylation changes as biomarkers of GDM. Instead, it supports the putative value of DNA methylation as a more stable and consistent marker of molecular injury across the developmental span. We found that DNA methylation profiles of the 42 placentas were sufficient to drive distinct clustering by maternal GDM disease status. When the methylation profile was built using DMCs with the ≥10% DNA methylation changes, male and female samples clustered by disease status. However, when smaller effect size changes (<10%) were also included in the methylation profile, only male samples clustered by disease status. This suggests that larger changes in DNA methylation (≥10%) are more consistent changes in GDM pregnancy, regardless of foetal gender, and these may more effectively predict disease status across samples and potentially across populations. The stronger clustering of male samples likely reflects the overrepresentation of male GDM samples in the dataset. A meta-analysis reported an increased risk of developing GDM in women carrying a male foetus[91]. However, the evidence is limited, with no mechanisms of action suggested to explain this potential relationship. The literature does report more consistently that male offspring from GDM pregnancies are at increased risk of negative perinatal outcomes [92,93]. However, this study was limited in its ability to address the sex effects of GDM due to the small sample size and underrepresentation of female GDM samples. If there are sex-specific effects, our bias in a greater number of male samples in case and control groups means our outcomes are likely enriched for effects on the male foetus.

This is the first study to report GDM-related changes in placental methylation at developmental genes involved in Wnt and cadherin signalling pathways. These pathways play key roles in embryogenesis, and Wnt signalling is necessary for placentation [94]. *WNT2* knockout models showed this gene is key to activating the Wnt signalling pathway in trophoblast cells to promote cell proliferation and migration and proper vascularization of the placenta [65,95]. The role of Wnt signalling in placental development and growth may have implications for neonatal outcomes as larger placentas are associated with foetal overgrowth [96]. The overrepresentation of these two pathways in analyses of genes adjacent to small (>10%) and larger (≥10%) effect size methylation changes has important implications for both placental and foetal development. Assessment of these changes with gene function and developmental phenotypes in a larger cohort will likely yield valuable insight into the mechanism by which GDM impairs placental and foetal development.

It is important to note that this study only reflects changes at a single time point in gestation (birth) and did not measure variation in the placental DNA methylation profile across gestation, as has been previously described [97]. Therefore, transient changes in DNA methylation would not be detected using our study design. In order to address this limitation, we adjusted for variation in gestational age at birth to reduce confounding by this variable. As reported by Novakovic et al. (2011), the inter-individual methylation variation in full-term placentas is driven by environmental factors like GDM status. Thus, the placental DNA methylation patterns at birth likely reflect, in part, an accumulation of adaptations in response to the GDM environment across gestation.

In addition to developmental pathway analyses, we characterized GDM effects at 14 candidate genes and defined consistent regional DNA methylation changes in the promoter/gene body of 11 of these genes. We propose that these DMRs may serve as both mechanistic targets and as valuable epigenetic biomarkers of GDM-associated placental molecular injury that could be developed into early detection markers of adverse outcomes of GDM pregnancies. For example, GDM-DMRs at insulin regulatory genes (*IRS1, ADORA2B, and PTPRN2*) are indicative of impaired insulin signalling in GDM placentas. Impaired insulin signalling is proposed to be a common signature of GDM. Multiple GWAS studies show that maternal polymorphisms in *IRS1* (a key protein involved in insulin signalling) are associated with predisposition to GDM [98–101]. Work in human fetoplacental endothelial cells showed that insulin is required to activate the IRS1/Pi3K/Akt pathway, a necessary step for increased angiogenesis during placentation [102]. *ADORA2B* has also been implicated in impaired placenta development and pre-eclampsia [103,104], and studies show increased *ADORA2B* expression in maternal leukocytes from GDM pregnancies [105].

This study identified relevant epigenetic perturbation at important placental development genes in placentas from lean and obese GDM pregnancies. Identification of these changes is an essential step towards understanding how the GDM environment during pregnancy affects foetal development and long-term offspring outcomes. It is also a critical step towards developing effective biomarkers for diagnoses and intervention testing. However, we only focused on DNA methylation and were likely underpowered with N = 42 to detect all potential epigenetic changes related to GDM. Further studies to integrate the role of variables such as sex, ethnicity, and genetic ancestry are necessary to elucidate interindividual differences in these outcomes. Therefore, this is only a partial survey of the impact of GDM on epigenetic regulation of the placenta. Future work is necessary to complete our understanding.

## Supplementary Material

Supplemental Material

## Data Availability

The datasets used during the study are currently available from the corresponding authors on reasonable requests. Data will be available within 6 months of the paper acceptance date at GEO through NCBI.
