# Supplementary material for: Gestational diabetes mellitus placentas exhibit epimutations at placental development genes
Source: Epigenetics. 2022 Aug 21;17(13):2157–77. doi: 10.1080/15592294.2022.2111751 (PMC9665155; doi:10.1080/15592294.2022.2111751)
Supplement: Supplemental Material [file KEPI_A_2111751_SM5198.zip › Supplementary/Meyrueix_SuppTable1_Epigenetics.docx]

| Supplemental Table 1. Association of GDM Status with placenta & birth outcomes in S1000. | | |
| --- | --- | --- |
|  | All Samples  (n = 42) | Male Samples  (n = 26) |
|  | Adjusted | Adjusted |
| **Placenta Outcomes** | β-coefficient (p-value) | β-coefficient (p-value) |
| Placenta weight^+^ (g) | -22.2 (0.601) | -44.6 (0.131) |
| Placenta efficiency^+^ (grams neonate/gram placenta) | -0.154 (0.756) | -0.105 (0.793) |
| **Birth Outcomes** | | |
| Birth weight (g) | -225.1 (0.113) | **-361 (0.024*)** |
| Birth head circumference (cm) | -0.778 (0.111) | -0.702 (0.222) |
| Birth length (cm) | -0.447 (0.731) | -1.77 (0.209) |
| Ponderal index (g x 100/cm^3^) | -0.091 (0.561) | 0.0134 (0.947) |

Linear regression adjusted for maternal age, gestational age, maternal BMI, and mode of delivery. Model that includes all samples includes sex of offspring. ^+^ denotes samples size of n = 33 for all Samples and n = 20 for male samples.
