# Supplementary material for: Gestational diabetes mellitus placentas exhibit epimutations at placental development genes
Source: Epigenetics. 2022 Aug 21;17(13):2157–77. doi: 10.1080/15592294.2022.2111751 (PMC9665155; doi:10.1080/15592294.2022.2111751)
Supplement: Supplemental Material [file KEPI_A_2111751_SM5198.zip › Supplementary/Meyrueix_SuppTable2_Epigenetics.docx]

| Supplemental Table 2. Top 10 Regulatory Elements. | | |
| --- | --- | --- |
| Transcription Factor | Regulatory Element Type | # of DMCs |
| EGR1 | Transcription Factor Binding Site | 956 |
| SMARCA4 | Transcription Factor Binding Site | 806 |
| RBL2 | Transcription Factor Binding Site | 728 |
| TFAP2C | Transcription Factor Binding Site | 387 |
| CTCF | Transcription Factor Binding Site | 205 |
| STAT1 | Transcription Factor Binding Site | 197 |
| CTCF | Regulatory Region | 185 |
| TRIM28 | Transcription Factor Binding Site | 180 |
| ETS1 | Transcription Factor Binding Site | 166 |
| RB1 | Transcription Factor Binding Site | 136 |

A total of 12,210 DMCs were compared to ORegAnno database and 4,942 were found to intersect with specific regulatory
