# Supplementary material for: Gestational diabetes mellitus placentas exhibit epimutations at placental development genes
Source: Epigenetics. 2022 Aug 21;17(13):2157–77. doi: 10.1080/15592294.2022.2111751 (PMC9665155; doi:10.1080/15592294.2022.2111751)
Supplement: Supplemental Material [file KEPI_A_2111751_SM5198.zip › Supplementary/Meyrueix_SuppTable3_Epigenetics.docx]

| Supplemental Table 3. Overrepresented PANTHER Pathways associated with DMCs ≥10% | | | | |
| --- | --- | --- | --- | --- |
| PANTHER Pathway | # of Genes | P-value | FDR | Fold Enrichment |
| Cadherin signaling pathway (P00012) | 15 | 8.61E-05 | 7.19E-03 | 3.37 |
| Wnt signaling pathway (P00057) | 20 | 8.07E-04 | 4.49E-02 | 2.32 |

A total of 559 genes were inputted into PANTHER and 539 of them mapped. Overrepresentation was calculated using a Fisher’s exact test and the False Discover Rate was calculated.
