# Supplementary material for: Gestational diabetes mellitus placentas exhibit epimutations at placental development genes
Source: Epigenetics. 2022 Aug 21;17(13):2157–77. doi: 10.1080/15592294.2022.2111751 (PMC9665155; doi:10.1080/15592294.2022.2111751)
Supplement: Supplemental Material [file KEPI_A_2111751_SM5198.zip › Supplementary/Meyrueix_SuppTable4_Epigenetics.docx]

| Supplemental Table 4 | |  |
| --- | --- | --- |
| Gene Name | Forward Primer Sequence | Reverse Primer Sequence |
| *EBF1* | 5’-TGCAAAGGAACACCAGGCAG-3’ | 5’-AGATCCGCAGCCCTTTTGAG-3’ |
| *GATA4* | 5’- CGAGATGGGACGGGTCACTA-3’ | 5’-GGCAGTTGGCACAGGAGAG-3’ |
| *HIST1H3E* | 5’-GTGAAGAAGCCCCATCGCTA-3’ | 5’-AGCTATTTCTCGCACCAGGC-3’ |
| *SMOC2* | 5’- TACGAGCAGCCGAAATGTGA-3’ | 5’-GTCCAGAACGCTGGTCAGAA-3’ |

Sequences for primers used in qRT-PCR experiments.
