# Supplementary material for: Gestational diabetes mellitus placentas exhibit epimutations at placental development genes
Source: Epigenetics. 2022 Aug 21;17(13):2157–77. doi: 10.1080/15592294.2022.2111751 (PMC9665155; doi:10.1080/15592294.2022.2111751)
Supplement: Supplemental Material [file KEPI_A_2111751_SM5198.zip › Supplementary/Supplemental Figure legends.docx]

**Supplemental Figure 1. Mean placental methylation levels on chr X.** Bar graph shows the average methylation level (± SD) for all queried CpGs on chr X for GDM vs nonGDM placenta samples stratified by sex.

**Supplemental Figure 2. PCA plots of methylation levels stratified by sex.** PCA plot of all queried CpGs (n = 989,582) for: (A) Male samples only; and (B) Female samples only. Ellipses indicate 95% confidence intervals for GDM (red) vs nonGDM (blue) samples.

**Supplemental Figure 3. Directional changes of GDM-related DMCs.** (A) The proportions of DMCs with gain (light blue, GOM) or loss (dark blue, LOM) of methylation in GDM placentas compared to nonGDM placentas. Data are shown for all DMCs, DMCs with small effect size changes (<10%), and DMCs with medium to large effect size changes (≥10%). (B) Proportion of LOM vs. GOM DMCs on each chromosome. (C) Proportion of LOM vs. GOM DMCs at each genic region.

**Supplemental Figure 4. Female sample methylation profiles at GDM-related DMCs using hierarchical clustering.** Hierarchical clustering of DMCs (n=12,210) in female samples only (n=16).
