# Supplementary figures and images for: Gestational diabetes mellitus placentas exhibit epimutations at placental development genes
Source: Epigenetics. 2022 Aug 21;17(13):2157–77. doi: 10.1080/15592294.2022.2111751 (PMC9665155; doi:10.1080/15592294.2022.2111751)

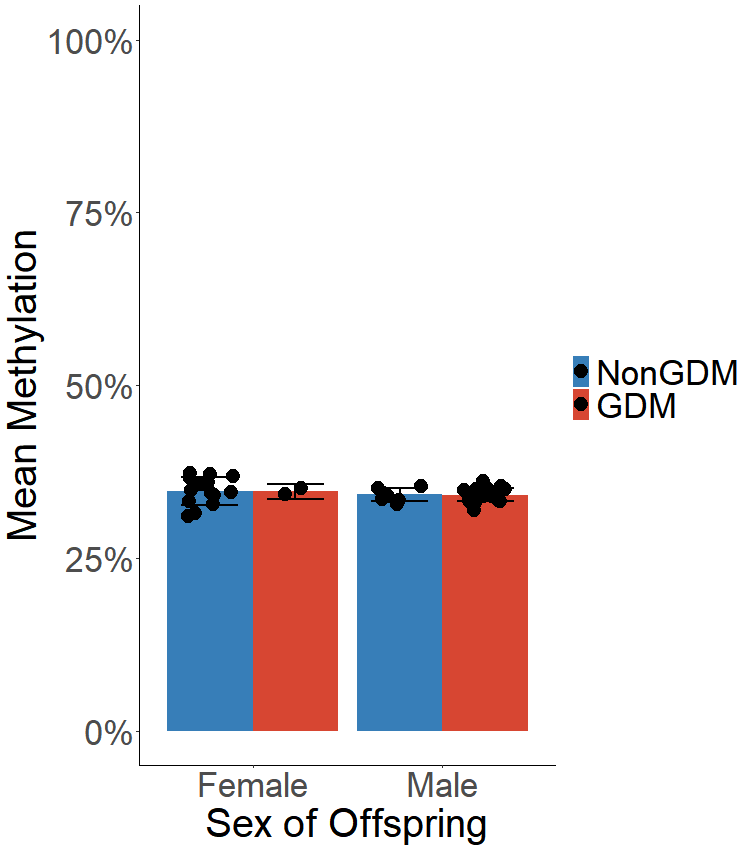

Supplement: Supplemental Material [file KEPI_A_2111751_SM5198.zip › Supplementary/Meyrueix_SuppFig1_Epi.docx]

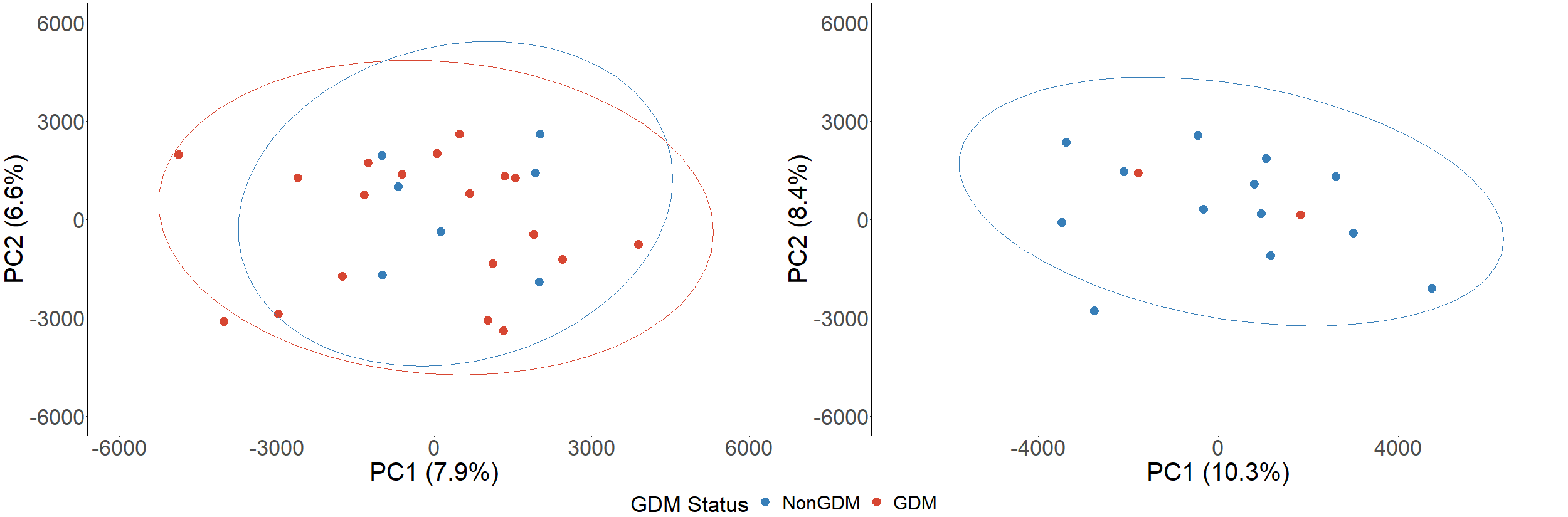


**A**

**B**

Supplement: Supplemental Material [file KEPI_A_2111751_SM5198.zip › Supplementary/Meyrueix_SuppFig2_Epi.docx]

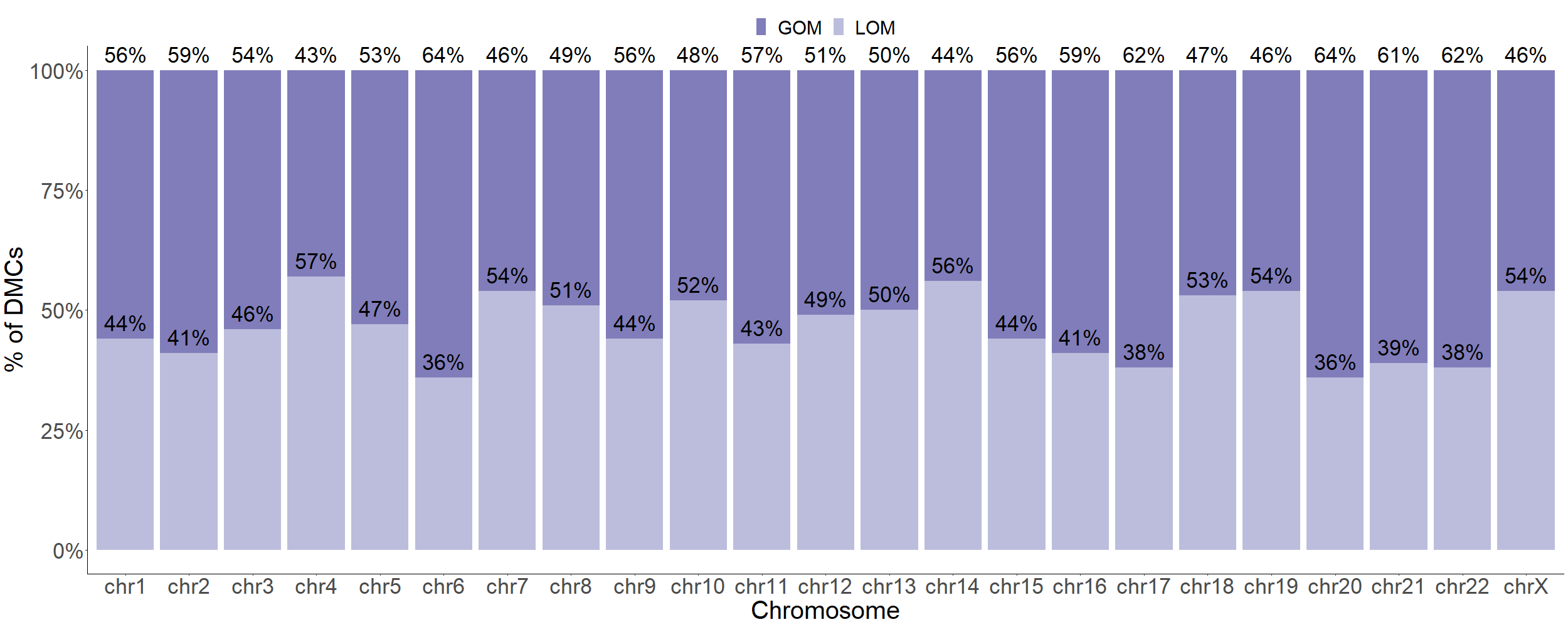

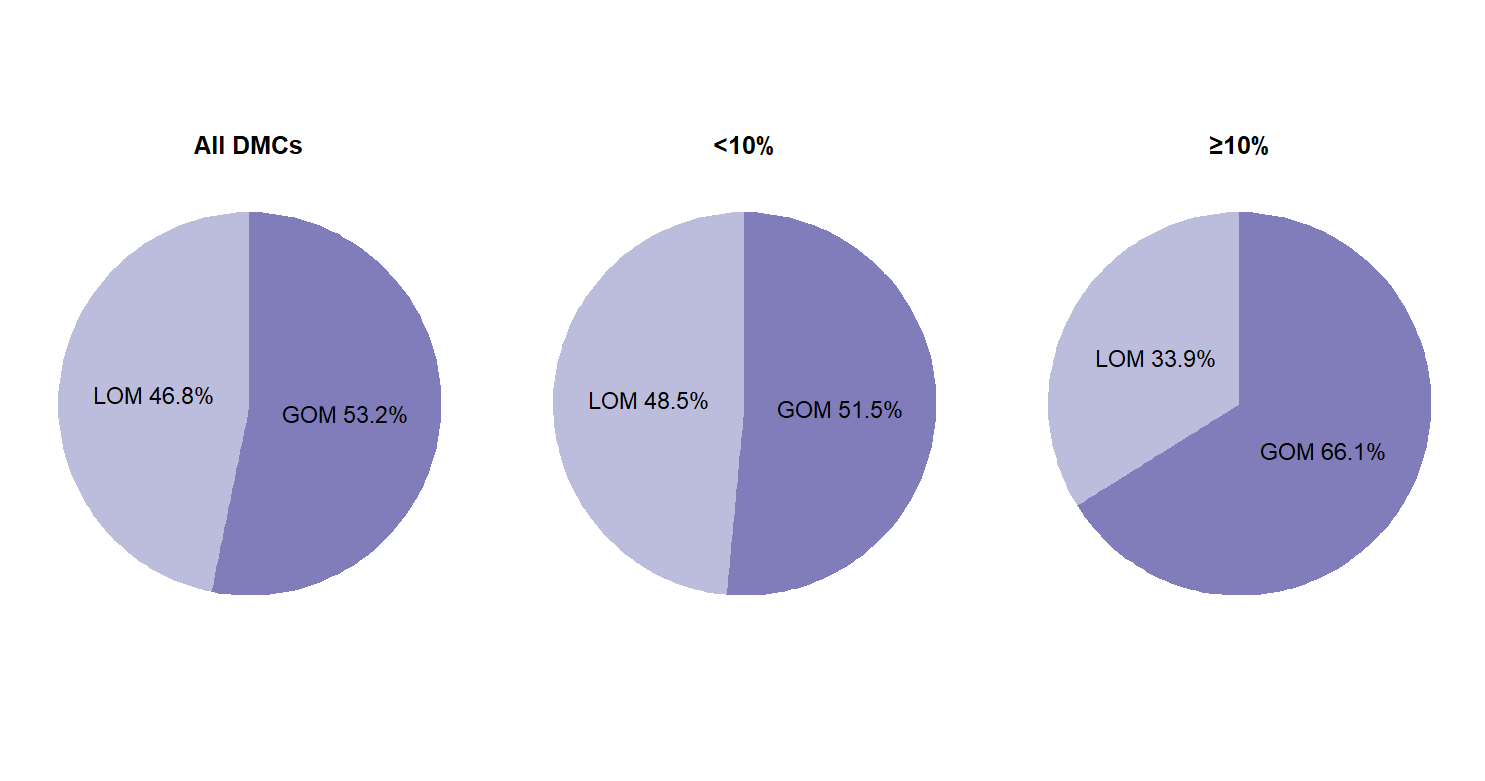


**A**

**B**


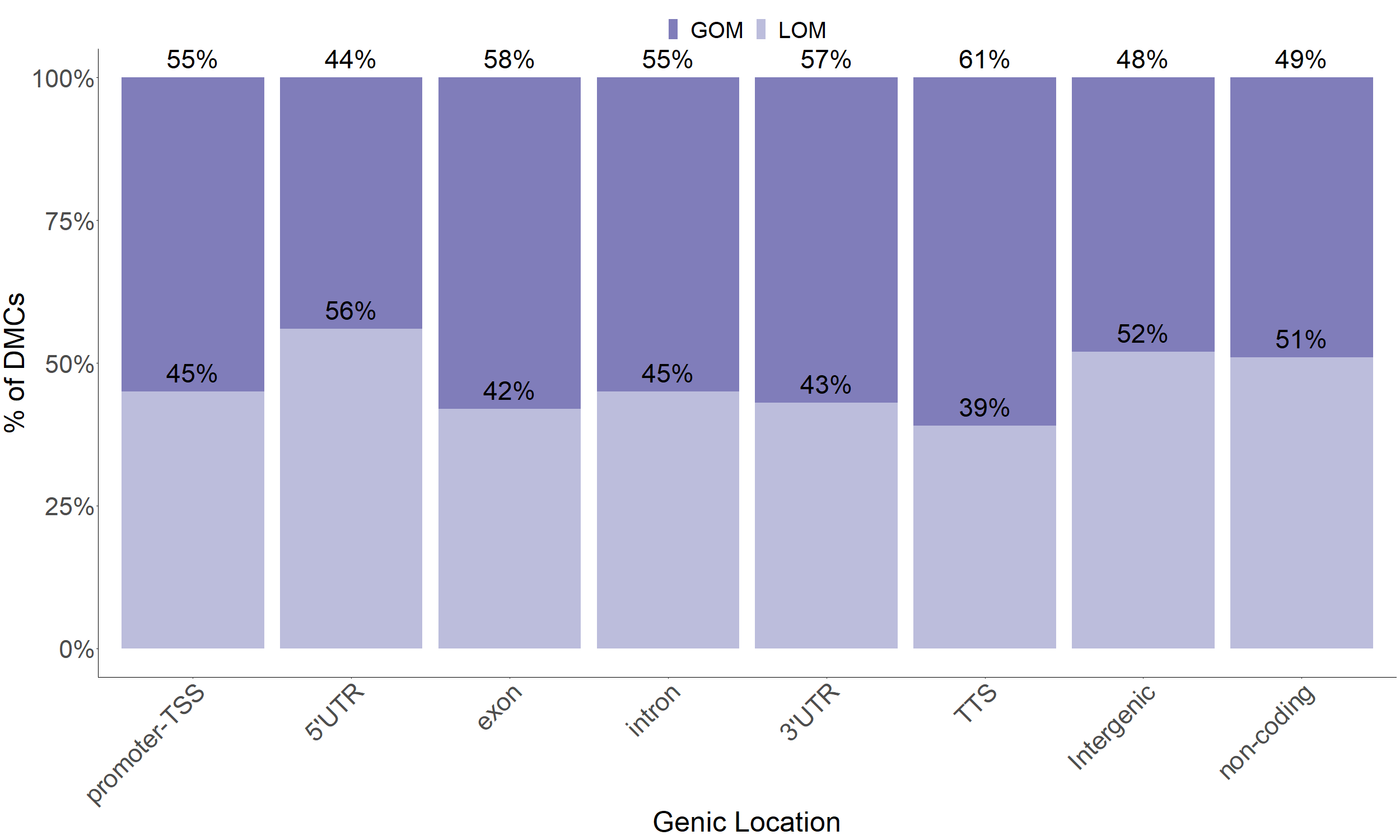


**C**

Supplement: Supplemental Material [file KEPI_A_2111751_SM5198.zip › Supplementary/Meyrueix_SuppFig3_Epi.docx]

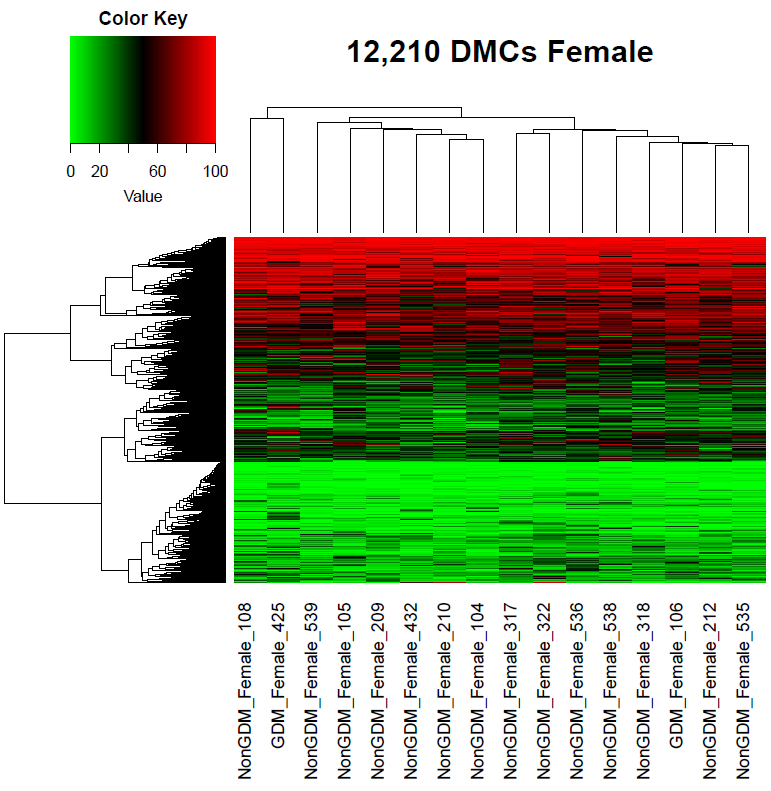

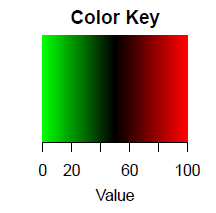


**Methylation%**

Supplement: Supplemental Material [file KEPI_A_2111751_SM5198.zip › Supplementary/Meyrueix_SuppFig4_Epi.docx]
